# Phytochemical profiling of strawberry fruits, and bioactive compounds from the same selected cultivar in human plasma during a medium-term consumption study

**DOI:** 10.1186/1753-6561-6-S3-P5

**Published:** 2012-06-01

**Authors:** Francesca Giampieri, Jose M Alvarez-Suarez, Sara Tulipani, Maurizio Battino

**Affiliations:** 1 Dipartimento di Scienze Cliniche Specialistiche, Facoltà di Medicina, Università Politecnica delle Marche, Ancona Italy; 2 Department of Nutrition and Food Science-CeRTA, Faculty of Pharmacy, University of Barcelona, Barcelona, Spain

## Background

It is known that a common denominator in the pathogenesis of most chronic diseases is the involvement of oxidative stress, related to the production by all aerobic organisms of reactive oxygen species and reactive nitrogen species. Also the modern and more complex theories on the role of-oxidative stress in biological processes confirms the importance of a balanced equilibrium between oxidant production and antioxidant defenses, for preserving health and longevity. In parallel, a growing body of epidemiological studies suggests a consistent association between the consumption of plant-based diets and a lower incidence of several chronic pathologies, including cancer, cardiovascular and neurodegenerative diseases.

## Materials and methods

The first aim of this work was to evaluate the nutritional quality of the strawberry cultivar *Sveva*, by measuring the total antioxidant capacity, total phenolic, total flavonoid, and total anthocyanin contents of the strawberry extract. In addition, the vitamin C content was quantified by HPLC, while anthocyanin pigments were identified using HPLC-DAD-ESI/MS.

The second aim of this work was to assess the effects of strawberry consumption on biomarkers of antioxidant status, in healthy subjects. A pilot study was carried out, and the 24 screened participants were involved in a medium-term strawberry consumption test. They were asked to consume 300 g of strawberry per day for 15 days, preferably at mid-morning and mid-afternoon between meals. The potential changes in plasma markers of antioxidant status were evaluated, by measuring the strawberry-dependent variation in plasma total antioxidants and in serum concentrations of Vitamin C and Acid uric by HPLC with electrochemical detection. Moreover, plasma protein carbonyl content, the most commonly used marker of protein oxidation, and plasma reactive oxygen metabolites (dROMs), one of the most used marker of hydroperoxides levels, were evaluated. Statistical analyses were performed using STATISTICA software and the data were analyzed with the Wilcoxon test. Results are expressed as mean values ± standard error. Differences at *p* < 0.05 were considered statistically significant.

## Results

The cultivar Sveva presented high nutritional attributes, especially regarding total phenolic, total flavonoid and vitamin C contents, and showed relevant and well-balanced antioxidant, micronutrient and phytochemical composition. In addition, the medium-term intake of strawberries resulted in a significant increase in plasma antioxidant capacity measured by TEAC, FRAP and BAP assays in all subjects, independently of the individual baseline levels. Finally, significant increases in ascorbate, but not in urate, concentrations were observed in serum, as well as a significant decrease in protein carbonyl content and in dROMS in plasma.

## Conclusions

Dietary antioxidants from fruit seem to play an important beneficial role in improving antioxidant defenses of the human body against the development of chronic diseases [1], so that the availability of high quality and nutritionally enriched fruit may be a useful tool when planning healthy diets. Strawberries contain many important dietary components including vitamins and minerals, and are a rich source of phytochemical compounds, which seem to have relevant biological activity on human health [1].
